# Strain-Compensated InGaAsP Superlattices for Defect Reduction of InP Grown on Exact-Oriented (001) Patterned Si Substrates by Metal Organic Chemical Vapor Deposition

**DOI:** 10.3390/ma11030337

**Published:** 2018-02-26

**Authors:** Ludovico Megalini, Simone Tommaso Šuran Brunelli, William O. Charles, Aidan Taylor, Brandon Isaac, John E. Bowers, Jonathan Klamkin

**Affiliations:** 1 Department of Electrical and Computer Engineering, University of California Santa Barbara, Santa Barbara, CA 93106, USA; ssuranbrunelli@umail.ucsb.edu (S.T.Š.B.); bowers@ece.ucsb.edu (J.E.B.); klamkin@ece.ucsb.edu (J.K.); 2 SUNY Polytechnic Institute, Albany, NY 12222, USA; wcharles@sunypoly.edu; 3 Materials Department, University of California Santa Barbara, Santa Barbara, CA 93106, USA; aidantaylor@ucsb.edu (A.T.); brandonjisaac@ucsb.edu (B.I.)

**Keywords:** InGaAsP strain compensated superlattices, MOCVD, hetero-epitaxy on Si, InP on Si, GaAs on Si

## Abstract

We report on the use of InGaAsP strain-compensated superlattices (SC-SLs) as a technique to reduce the defect density of Indium Phosphide (InP) grown on silicon (InP-on-Si) by Metal Organic Chemical Vapor Deposition (MOCVD). Initially, a 2 μm thick gallium arsenide (GaAs) layer was grown with very high uniformity on exact oriented (001) 300 mm Si wafers; which had been patterned in 90 nm V-grooved trenches separated by silicon dioxide (SiO_2_) stripes and oriented along the [110] direction. Undercut at the Si/SiO_2_ interface was used to reduce the propagation of defects into the III–V layers. Following wafer dicing; 2.6 μm of indium phosphide (InP) was grown on such GaAs-on-Si templates. InGaAsP SC-SLs and thermal annealing were used to achieve a high-quality and smooth InP pseudo-substrate with a reduced defect density. Both the GaAs-on-Si and the subsequently grown InP layers were characterized using a variety of techniques including X-ray diffraction (XRD); atomic force microscopy (AFM); transmission electron microscopy (TEM); and electron channeling contrast imaging (ECCI); which indicate high-quality of the epitaxial films. The threading dislocation density and RMS surface roughness of the final InP layer were 5 × 10^8^/cm^2^ and 1.2 nm; respectively and 7.8 × 10^7^/cm^2^ and 10.8 nm for the GaAs-on-Si layer.

## 1. Introduction

The direct growth of InP and GaAs on Silicon (Si) is of strong interest for the fabrication of monolithically integrated lasers in silicon photonics (SiPh) and, more generally, to realize opto-electronic integrated circuits (OEICs). Large-scale manufacturing would prefer monolithic solutions to techniques like flip-chip and wafer bonding, although these are currently more mature [1,2]. In particular, hetero-epitaxy by MOCVD is attractive due to the ability of this technique to grow a broad range of compounds for photonics and electronics devices in large-scale and high-yield [3].

However, the direct heteroepitaxy of InP/GaAs on Si is extremely challenging: the large lattice mismatches between InP, GaAs, and Si $\left(\varepsilon_{InP/Si}\approx {8\%,\;\varepsilon }_{GaAs/Si}\approx 4\%\right)$, their different polarities and thermal expansion coefficient cause the formation in high density of defects, including anti-phase domains (APDs), stacking faults, twins, threading, and misfit dislocations, which typically exceed 1 × 10^9^/cm^2^ [4] and as a consequence they reduce significantly the performance and reliability of the fabricated devices. Therefore defect engineering techniques have been used to improve the epitaxial film quality including growth of Ge [5], GaAs [6], or GaP [7] thin buffer layer either on exact oriented (001) or off-cut Si wafers [8], use of thermal cycling both during the different layers growth and at the end of the final epitaxial stack [9], selective area growth (SAG) [10] often combined with aspect ratio trapping (ART) [11,12,13,14], strain engineering by growth of graded buffer layers like InAlAs [15], and strained layer superlattices (SLSLs) [16], typically used as threading dislocation (TDs) filtering. Indeed, these last two techniques tend to favor TD movement and interaction [17], which can cause TD annihilation or movement toward the edge of the wafer, ultimately resulting in wide defect-free regions of the epitaxial structure. Strained layer superlattices have been extensively studied and successfully employed in GaAs-on-Si growth [18,19,20,21,22], GaN-on-sapphire [23,24], and GaN-on-Si [25,26], while their use in InP-on-Si has been rather limited [27].

In this work, we propose the use InGaAsP strain-compensated superlattices as an additional tool to reduce the defect density in InP-on-Si. To the best of our knowledge, this is the first report showing strain compensation using InGaAsP alloys for both the tensile and compressive layers. The use of quaternary compounds is particularly interesting because it allows us to independently control the strain and the composition of each strained layer. Moreover, the InGaAsP material system does not suffer of the typical growth issues of III-nitride compounds so that all the compressive and tensile layers can be grown at their optimum temperature without the need of long waiting time for temperature ramp up and cool down, as is the case in the InGaN/AlGaN system [28].

## 2. Materials Growth 

**Figure 1:**
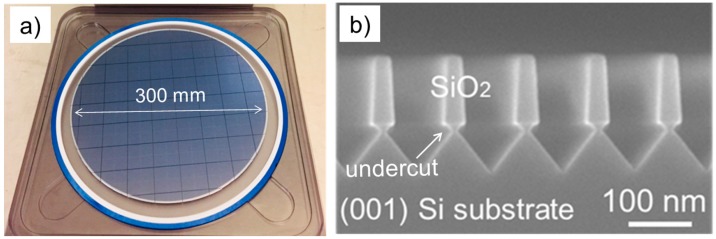
(**a**) Photo illustrating the 300 mm exact (001) Si wafer used a substrate for the III–V growth; (**b**) cross-section SEM image of the Si wafer patterning.

The epitaxial structure was grown entirely by MOCVD. Initially, a 2 μm thick GaAs layer was grown on a 300 mm patterned Si wafer (Figure 1a). The pattern consisted of 90 nm wide trenches separated by 65 nm wide SiO_2_ stripes. V-grooves were formed by etching the Si trenches with dilute potassium hydroxide (KOH) at 70 °C such that the growth was initiated on {111} Si surface to avoid anti-phase domains formation (APDs) [29]. Additionally, a small undercut was formed at the Si/SiO_2_ interface to exploit the defect necking mechanism and reduce most of the stacking fault propagation from the Si device layer into the III–V layer (Figure 1b) [30,31].

### 2.1. GaAs on Si

A GaAs buffer layer was initially grown on Si (GaAs-on-Si) and used as a platform for the subsequent InP-based structure. A patterned Si wafer was loaded into an Aixtron Crius-R reactor that can accommodate one 300 mm silicon wafer and it was exposed to vaporized hydrofluoric acid (HF) in a pre-clean reactor chamber to remove the oxide between the trenches. Then the wafer was transferred to the reactor growth chamber where it was heated to 900 °C for 10 min in a high pressure H_2_ to remove the native oxide and to further clean the wafer surface. After exposing the wafer surface to tertiarybutylarsine (TBAs), a thin (~7 nm) nucleation layer was grown at a temperature of 400 °C using trimethylgallium (TMGa) and tertiarybutylarsine (TBAs). The TBAs flash and the low temperature layer help to accommodate the lattice mismatch between the III–V and the Si substrate and the III–V layers [32]. Then a high quality GaAs layer was grown in a temperature range of 600 °C to 650 °C using TMGa and arsine (AsH_3_) with a V/III ratio of 100. The wafer was annealed in a temperature range of 650 °C to 750 °C in an arsenic rich ambient for 20 min. 

### 2.2. InP and Strain Compensated InGaAsP Superlattice on Si

**Figure 2:**
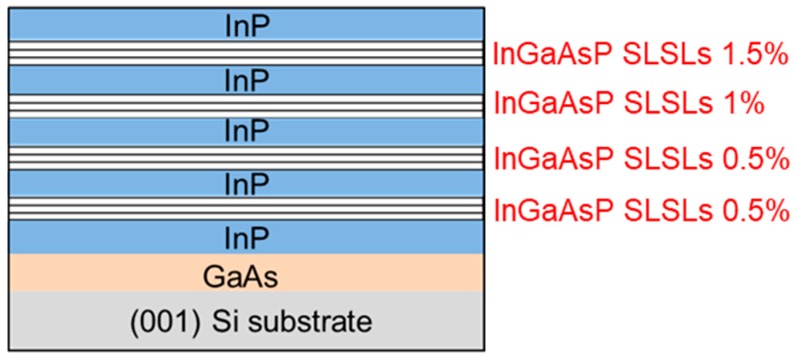
Schematic of the epitaxial stack.

The 300 mm GaAs-on-Si wafer was diced in 2 × 2 cm^2^ pieces to allow loading into a different Aixtron MOCVD reactor. Following an oxide desorption step at 550 °C under TBAs flow, the temperature was decreased to 430 °C to deposit a thin (~20 nm) low-temperature InP nucleation layer to accommodate the lattice mismatch between InP and GaAs ($\varepsilon_{InP/GaAs}\approx 4\%$). The V/III ratio was 628. Next, the temperature was increased to 610 °C and the InP growth rate was progressively increased until a growth of 3.7 Å/s was reached [33]. Trimethylindium (TMIn) and tertiarybutyl phosphine (TBP) were used as group III and V precursors, respectively. After 600 nm thick InP was grown, four ${In}_{x_{1}}{Ga}_{1-x_{1}}{As}_{y_{1}}P_{1-y_{1}}/{In}_{x_{2}}{Ga}_{1-x_{2}}{As}_{y_{2}}P_{1-y_{2}}$ strain compensated superlattices were inserted. The superlattices were separated by a 300 nm thick InP spacer. Each layer of the first two superlattices had a strain of |0.5|%, while the third and fourth superlattices were made of layers having each a strain of |1|% and |1.5|%, respectively. The epitaxial stack ended with a 600 nm InP cap layer. Finally, an annealing under TBP flow at 610 °C for 10 min was carried out to favor the InP surface smoothing [34]. The reactor pressure was maintained at 350 Torr during the all growth. The final epitaxial structure is shown in Figure 2.

## 3. Materials Characterization

The epitaxial layers were characterized by a variety of techniques. First of all, we verified the uniformity of the GaAs layer grown on the 300 mm patterned Si wafer, being the growth of high quality materials with high uniformity while maintaining a high throughput one of the III–V on Si monolithic integration key requirement. All these features are typical of the MOCVD growth technique. Later, we characterized the InP-on-GaAs-on-Si epitaxial quality, in particular, we studied the design and the impact of the InGaAsP superlattice on reducing the defect density.

### 3.1. GaAs on Si

The uniformity and the quality of the GaAs layer were analyzed by X-ray diffraction using a PANalytical MRD PRO high resolution X-ray diffractometer (XR-XRD, PANalytical solutions, Almelo, Netherland) with a Cu kα1 (1.5405 Å) source operated at 40 kV voltage and 45 mA current. The (004) ω/2θ s triple axis scan clearly shows both the GaAs and the Si peak at 32.95° and 34.67°, respectively (Figure 2a) while a double axis configuration was used for on axis (004) ω-rocking curves. The Full width half maximum (FWHM) was 167 arcsec, with a standard deviation of ~7% across the 300 mm Si wafers. 

**Figure 3:**
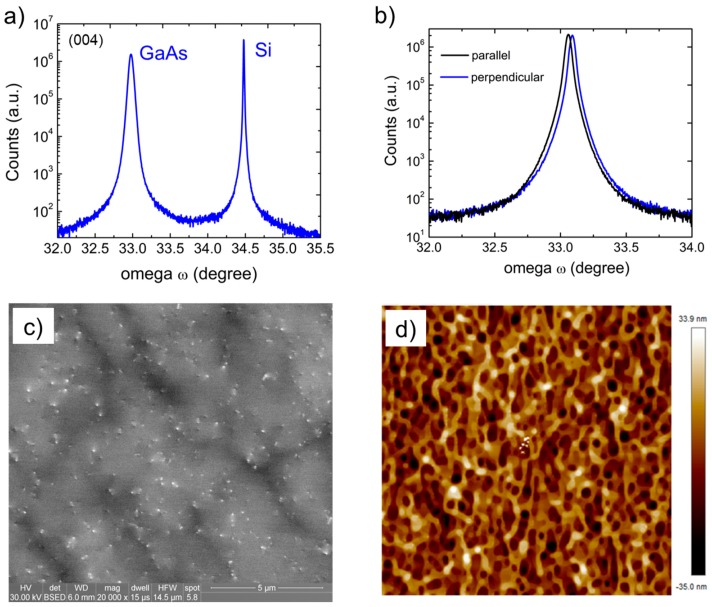
(**a**) ω-2θ scan; (**b**) w rocking curve; (**c**) ECCI; and (**d**) AFM scan of GaAs on Si epitaxial structure. AFM scan is over an area 5 × 5 μm^2^.

The small difference in FWHM of the scan perpendicular vs. parallel to the trench orientation suggests a slight difference in the defect distribution. This has been attributed to the aspect ratio trapping technique [35]. The threading dislocation density was estimated to be 8.5 × 10^7^/cm^2^ by electron contrast channeling imaging (ECCI) and the RMS roughness was 10.0 nm as measured by atomic force microscopy (AFM, Bruker Nano, Billerica, MA, USA) as shown in Figure 3c,d, respectively. The relatively high roughness is mainly attributed to the desorption of arsenic during the high temperature anneal step.

### 3.2. InP-on-GaAs-on-Si 

**Figure 4:**
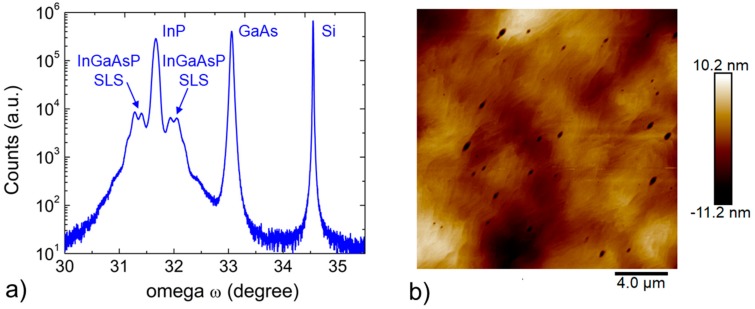
(**a**) ω-2θ scan and (**b**) AFM scan of the final epitaxial structure.

The InP and the ${In}_{x}{Ga}_{1-x}{As}_{y}P_{1-y}$ strain compensated superlattices grown on the GaAs-on-Si were preliminarily characterized by X-ray diffraction. Figure 4a show the (004) ω/2θ s triple axis configuration of the final epitaxial structure. The ${In}_{x}{Ga}_{1-x}{As}_{y}P_{1-y}$ superlattices appear as two shoulders symmetric to the InP peak. The RMS roughness of the final structure is 1.2 nm, as shown in Figure 4b. 

### 3.3. InGaAsP Strain Compensated Superlattice 

**Figure 5:**
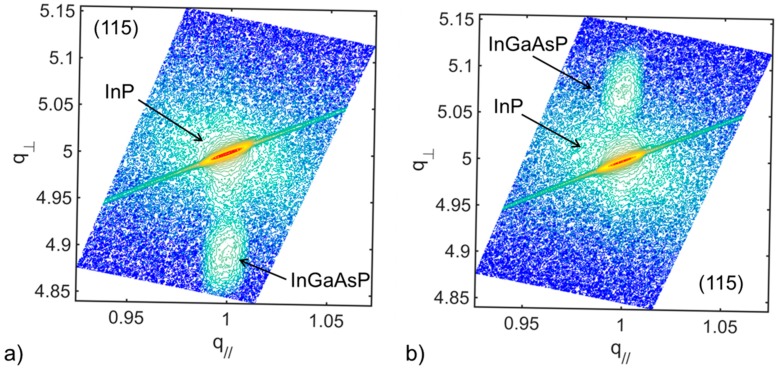
RSM map of (**a**) 1% compressive and (**b**) 1% tensile strained InGaAsP layer on bulk InP.

Four strain compensated superlattices (SC-SLs) were inserted in the InP layer. Each superlattice consisted of 4× (changed, pls confirm) pairs of ${In}_{x_{1}}{Ga}_{1-x_{1}}{As}_{y_{1}}P_{1-y_{1}}/{In}_{x_{2}}{Ga}_{1-x_{2}}{As}_{y_{2}}P_{1-y_{2}}$. The composition and thickness of each layer had been previously characterized on bulk InP substrate such that each compressive and tensile strained layer was grown coherently to InP. Figure 5a,b show the reciprocal space mapping (RSM) around (115) off-axis Bragg reflection of the 1% compressive strained ${In}_{x_{1}}{Ga}_{1-x_{1}}{As}_{y_{1}}P_{1-y_{1}}$ and 1% tensile ${In}_{x_{2}}{Ga}_{1-x_{2}}{As}_{y_{2}}P_{1-y_{2}}$ layer.

## 4. Discussion

Strain has been used as a powerful tool in the design of multi-quantum-well (MQW) active region of high-performance laser [36]. In particular, strain compensation has proven to be advantageous to devices reliability [37] by improving their thermal and structural stability [38]. Strain compensated structures emitting at 1550 nm have manifested stronger photoluminescence and narrower FHWM spectra [39] and 1.06 μm optical modulators have shown improved performance [40].

Strain compensated superlattices are also beneficial to hetero-epitaxial growth because the driving force for the generation of misfit dislocation is reduced compared to the uncompensated structure [41,42].

In this work, the strain of each superlattice was obtained by alternating ${In}_{x}{Ga}_{1-x}{As}_{y}P_{1-y}$ layers such that their strain was equal in magnitude and opposite in sign in order to obtain strain compensation. The layer thickness was kept ~15% below the Matthew-Blakeslee critical thickness *h*_c_ for each specific strain in order to avoid the layer relaxation. The biaxial strain component $\varepsilon_{xy}$ of a strained InGaAsP layer grown on a relaxed InP layer perpendicular to the growth direction is defined as

(1) $$\varepsilon_{xy}=\frac{a_{InP}-a_{In_{x}Ga_{1-x}As_{y}P_{1-y}}}{a_{In_{x}Ga_{1-x}As_{y}P_{1-y}}}$$

and the average lattice constant $\langle a_{pair}\rangle$ of each strained layer pair is

(2) $$\langle a_{pair}\rangle =\frac{t_{compressive}\cdot a_{In_{x_{1}}Ga_{1-x_{1}}As_{y_{1}}P_{1-y_{1}}}+t_{tensile}\cdot a_{In_{x_{2}}Ga_{1-x_{2}}As_{y_{2}}P_{1-y_{2}}}}{t_{compressive}+t_{tensile}}$$

where $t_{compressive}$ ($t_{tensile}$) is the thickness of the compressive (tensile) layer of composition $a_{In_{x_{1}}Ga_{1-x_{1}}As_{y_{1}}P_{1-y_{1}}}$($a_{In_{x_{2}}Ga_{1-x_{2}}As_{y_{2}}P_{1-y_{2}}})$, respectively.

Thus the average strain component of each superlattice is estimated as

(3) $$\varepsilon_{xy}=\frac{a_{InP}-\sum_{1}^{4}\langle a_{pair}\rangle }{\sum_{1}^{4}\langle a_{pair}\rangle }$$

**Figure 6:**
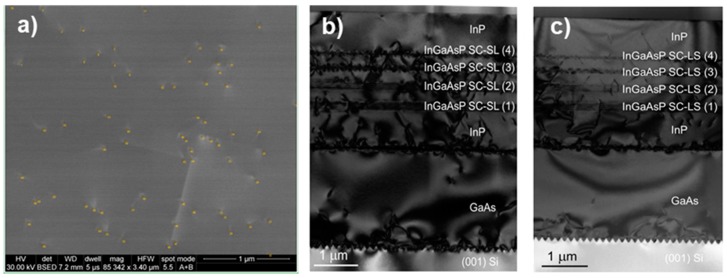
(**a**) Plain view ECCI image of the final epitaxial structure; (**b**,**c**) TEM cross-section TEM images showing the epitaxial stack. The SC-SLs are clearly visible and they are ordered 1–4 as listed in Table 1.

The final structure presents a defect density of ~5 × 10^8^/cm^2^ as shown in Figure 6a. The low RMS roughness value of 1.2 nm suggests that the strain compensated superlattices have also contributed to smoothing the relatively rough surface of the initial GaAs-on-Si epitaxial structure. Moreover, they have helped to filter most of the defects as illustrated in the TEM images of Figure 6b,c.

**Figure 7:**
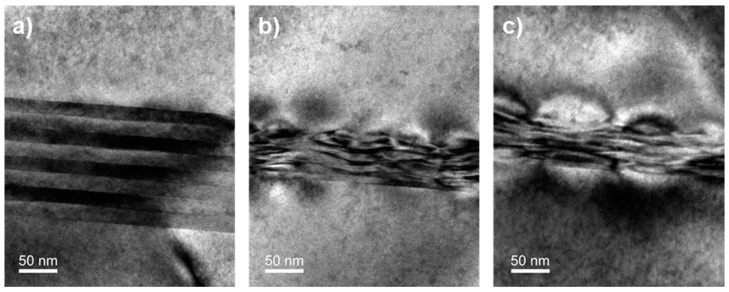
Dark field TEM images of the (**a**) 0.5%; (**b**) 1%; and (**c**) 1.5% strain compensated superlattices.

TEM images have also shown that the interfaces of the compensated strain superlattices are very sharp in the case of the first two superlattices (Figure 7a), differently from the third and fourth superlattices (Figure 7b,c). This can be attributed to the extremely challenging task of growing a quaternary layer. Indeed, the flow of each metallorganic source needs to be monitored very carefully in order to control precisely both the layer thickness and composition. During the first two superlattices growth (absolute strain = |0.5|%), only the TBAs flow was changed between the tensile and compressive layer and the others flows were maintained constant. On the other hand, in the case of the third and fourth superlattices, both the TBAs and the TMGa were changed in order to achieve a higher strain (absolute strain = |1|% and |1.5|%, respectively). It is worth noting that the higher is the layer strain, the thinner is the grown layer. During all the superlattice growths, a 1.5 s pause was used between the compressive and tensile layer in order to evacuate the reactor chamber of residual gases.

Further improvement is expected by optimization of the strain of each layer of superlattices, in particular of the number of superlattice periods and their thickness, and of the growth conditions of the GaAs buffer layer.

## 5. Conclusions

In this work, we have presented the growth by MOCVD of a smooth and high quality InP on exact (001) Si wafers using GaAs as buffer layer. Compensated strain InGaAsP superlattices were used as dislocation filters and they have shown to be helpful to reduce the propagation of threading dislocations from the substrate into the active region of the device. This approach is promising to grow InP virtual substrates directly on 300 mm exact (001) Si wafer grow by MOCVD which is necessary to monolithically integrate the next generation of photonic devices on Si with high-yield and high-throughput.

## Figures and Tables

**Table 1:** Summary of the parameters of the superlattices used in this study.

Superlattice	Composition	Strain (%)	PL (nm)	*h*_c_ (Å)
1st–c	In_69.7_Ga_30.3_As_50_P_50_	+0.5	1164.3	244.6
1st–t	In_69.7_Ga_30.3_As_80.9_P_19.1_	−0.5	1569.7	242.1
2nd–c	In_69.7_Ga_30.3_As_50_P_50_	+0.5	1164.3	244.6
2nd–t	In_69.7_Ga_30.3_As_80.9_P_19.1_	−0.5	1569.7	242.1
3rd–c	In_94.1_Ga_5.9_As_44.2_P_55.8_	+1.0	1302.8	86.2
3rd–t	In_63.2_Ga_36.8_As_49_P_51_	−1.0	1105.9	94.7
4th–c	In_52_Ga_48_As_58_P_42_	+1.5	1104.1	64.2
4th–t	In_90_Ga_10_As_68.7_P_31.3_	−1.5	1649.2	60.8

Note: PL is the photoluminescence wavelength of the layer, *h*_c_ is the critical thickness and is estimated using [17].
